# EZH2 as a Prognostic Factor and Its Immune Implication with Molecular Characterization in Prostate Cancer: An Integrated Multi-Omics in Silico Analysis

**DOI:** 10.3390/biom12111617

**Published:** 2022-11-02

**Authors:** Tian-Qi Du, Ruifeng Liu, Qiuning Zhang, Hongtao Luo, Zhiqiang Liu, Shilong Sun, Xiaohu Wang

**Affiliations:** 1 The First School of Clinical Medicine, Lanzhou University, Lanzhou 730000, China; 2 Institute of Modern Physics, Chinese Academy of Sciences, Lanzhou 730000, China; 3 Graduate School, University of Chinese Academy of Sciences, Beijing 101408, China

**Keywords:** prostate cancer, EZH2, bioinformatics, prognosis, immunotherapy

## Abstract

Prostate cancer (PCa) is a type of potentially fatal malignant tumor. Immunotherapy has shown a lot of potential for various types of solid tumors, but the benefits have been less impressive in PCa. Enhancer of zeste homolog 2 (EZH2) is one of the three core subunits of the polycomb repressive complex 2 that has histone methyltransferase activity, and the immune effects of EZH2 in PCa are still unclear. The purpose of this study was to explore the potential of EZH2 as a prognostic factor and an immune therapeutic biomarker for PCa, as well as the expression pattern and biological functions. All analyses in this study were based on publicly available databases, mainly containing Cancer Genome Atlas (TCGA), Gene Expression Omnibus (GEO), UCSCXenaShiny, and TISIDB. We performed differential expression analysis, developed a prognostic model, and explored potential associations between EZH2 and DNA methylation modifications, tumor microenvironment (TME), immune-related genes, tumor mutation burden (TMB), tumor neoantigen burden (TNB), and representative mismatch repair (MMR) genes. We also investigated the molecular and immunological characterizations of EZH2. Finally, we predicted immunotherapeutic responses based on EZH2 expression levels. We found that EZH2 was highly expressed in PCa, was associated with a poor prognosis, and may serve as an independent prognostic factor. EZH2 expression in PCa was associated with DNA methylation modifications, TME, immune-related genes, TMB, TNB, and MMR. By gene set enrichment analysis and gene set variation analysis, we found that multiple functions and pathways related to tumorigenesis, progression, and immune activation were enriched. Finally, we inferred that immunotherapy may be more effective for PCa patients with low EZH2 expression. In conclusion, our study showed that EZH2 could be a potentially efficient predictor of prognosis and immune response in PCa patients.

## 1. Introduction

Prostate cancer (PCa) is a type of potentially fatal malignant tumor. In 2020, PCa was the second most prevalent malignancy in men worldwide, with an expected 1.41 million new cases annually, just 20,000 cases less than lung cancer [1]. The estimated incidence rate of PCa across all ages (age-standardized rate, ASR) was 31 per 100,000 males, with a cumulative risk of 3.9% over a lifetime. With an estimated 375,304 deaths in 2020, PCa ranked as the fifth leading cause of cancer-related deaths worldwide, whose all-age mortality ASR was predicted to be 7.7 [2]. Despite the significant contribution of early surveillance and drug androgen deprivation therapy (ADT), surgical resection, radiotherapy and systemic chemotherapy to reduce morbidity and mortality, their prognosis, especially progression-free survival (PFS), remains unsatisfactory due to their metastatic, recurrent and heterogeneous nature. Accordingly, a greater comprehension of the biological behavior of PCa oncogenesis and progression will boost the development of more innovative and effective methods for the clinical diagnosis and treatment of patients.

Over the past few years, cancer immunotherapy has no longer been limited to understanding the interaction between cancer and the immune system but can also be employed as a predictor of tumor progression and clinical outcome. Thus, the amount of immunotherapy trials for numerous solid tumors has increased dramatically. Immunotherapy has shown a lot of potential for various types of solid tumors. However, it takes a considerably longer period to trigger an antitumor immune response since the progression of PCa is relatively slow and the clinical benefits have been limited [3]. This is primarily due to the fact that PCa is a “cold” tumor with very limited T-cell infiltration and a very limited response to immune checkpoint blockade (ICB) therapy with a single agent [4]. Furthermore, information regarding synergy with immune checkpoints in PCa is still minimal. Hence, it is imperative to explore new targets through the analysis of gene expression and the investigation of its associations with clinical prognosis and tumor immunity.

Enhancer of zeste homolog 2 (EZH2) is one of the three core subunits of the polycomb repressive complex 2 (PRC2) that has histone methyltransferase (MTase) activity. By catalyzing histone H3 lysine 27 trimethylation (H3K27me3), EZH2 induces chromatin condensation, which in turn leads to the epigenetic silencing of targeted genes [5]. It has been demonstrated that EZH2 is overexpressed in a variety of malignancies and is associated with a poor prognosis and that downregulating EZH2 expression can inhibit tumor cell proliferation, migration, and invasion [6,7,8,9,10,11]. In addition, we noticed that recent studies indicate that inhibiting EZH2 enhances antigen presentation, immune cell activation, and tumor sensitivity to PD-1 blockade [12,13]. In this study, we intend to conduct a comprehensive data mining analysis integrating different public databases to evaluate EZH2 expression and alterations, establish a prognostic model using EZH2 for PCa, and determine the potential of EZH2 as a synergistic target for immunotherapy and its biological function in PCa.

## 2. Materials and Methods

### 2.1. Acquirement of PCa Expression Profiles and Analysis of Gene Differential Expression

mRNA-sequencing data (level 3, FPKM normalized) and clinical information of PCa and corresponding normal tissues were downloaded from the official website of the Cancer Genome Atlas (TCGA, https://portal.gdc.cancer.gov, accessed on 19 June 2022). The log2 (transcripts per million (TPM) + 1) transformation was applied to all expression data for normalization and preprocessing. In this study, we extracted EZH2 expression data from 499 PCa tissues and 52 matched adjacent normal prostate tissues. Analysis of the differential expression levels was performed by using R software (Auckland, New Zealand) and the package “ggplot2”. Moreover, we validated the differential mRNA expression of EZH2 at the pan-cancer level in the Pan-Cancer Analysis of Whole Genomes (PCAWG) project using UCSCXenaShiny v1.1.8 (https://hiplot-academic.com/advance/ucsc-xena-shiny accessed on 19 June 2022), an online tool based on the University of California Santa Cruz (UCSC) Xena database [14].

### 2.2. Immunohistochemistry Analysis of EZH2

The Human Protein Atlas database (HPA, http://www.proteinatlas.org, accessed on 21 June 2022) which includes protein data of tumor and normal clinical samples, was used to analyze the expression pattern of EZH2 at the protein level [15]. Photomicrographs of EZH2 immunohistochemistry (IHC) staining in PCa and corresponding normal tissues were obtained from the pathology section and the tissue section, respectively. All the tumor tissues and normal tissues were processed with EZH2 antibody CAB009589 (Mouse, NCL-L-EZH2; Leica Biosystems, Deer Park, IL, USA). Image-Pro Plus 6.0 software (Media Cybernetics, Rockville, MD, USA) was used to measure the mean optical density (MOD) values of IHC.

### 2.3. The Prognostic Value of EZH2 in PCa

In order to categorize patients into high- and low-expression groups, the median EZH2 expression value among the 499 TCGA-PRAD patients was used as the cut-off value. We plotted overall survival (OS), PFS, and disease-specific survival (DSS) Kaplan-Meier (K-M) curves to analyze the different survival outcomes by expression level using R-package “survminer” and “survival”. Statistical significance was evaluated by using the log-rank test. Diagnostic receiver operating characteristic curve (ROC) analysis and time-dependent survival ROC as well as their area under the curve (AUC) values were performed using R-package “pROC” and “timeROC”, respectively. Both univariate and multivariate cox hazard regression analyses were employed to determine independent prognostic factors using R-package “forestplot” and “survival”. Subsequently, a nomogram prognostic model was developed to predict the probabilities of different survival times by using R-package “rms”.

A calibration curve was established using the bootstrap method, which allowed for the visualization of the variance between predicted and actual probabilities, conducted by R-package “rms” and “survival”. Decision curve analysis (DCA) was also plotted by using R-package “rmda”.

### 2.4. Methylation and Genetic Alteration Analysis

MEXPRESS database and Illumina human methylation450 data were used to analyze the association of EZH2 expression with the methylation level of CpG. Tumor IMmune Estimation Resource (TIMER, https://cistrome.shinyapps.io/timer/ (accessed on 26 June 2022)) is a web server for comprehensive analysis of tumor immunological, clinical, and genomic features, which was used to analyze the association of EZH2 expression with common DNA methyltransferases (DNMTs) [16]. The cBioPortal for Cancer Genomics (https://www.cbioportal.org, accessed on 26 June 2021) is an open-access resource for interactive exploration of multidimensional cancer genomics data sets, which were used to analyze EZH2 alteration frequency, mutation type, and copy number alteration (CNA) [17,18]. To determine the survival differences between those with and without the EZH2 genetic alteration group, K-M survival curves of OS and PFS were also conducted.

### 2.5. Differentially Expressed Genes and Prediction of Upstream miRNAs and lncRNAs of EZH2

Firstly, the patients from the TCGA-PRAD cohort were classified into two groups according to the median EZH2 expression value. Differentially expressed genes (DEGs) were confirmed by differentiation analysis between the high- and low-expression groups using R-package “DESeq2”. DEGs were defined as significant when both of the following criteria were met: (1) false discovery rate (FDR) adjusted *p*-value < 0.05; (2) |log2 (fold change (FC))|≥ 2.5.

The Encyclopedia of RNA Interactomes (ENCORI, http://starbase.sysu.edu.cn/, accessed on 30 June 2022) is a free online tool for determining cellular interactions between microRNAs (miRNAs), noncoding RNAs (ncRNAs), mRNAs, and RNA binding proteins (RBPs) from numerous high-throughput multidimensional sequencing data [19]. ENCORI is configured with various relevant prediction tools, including PITA, RNA22, miRmap, DIANA-microT, miRanda, PicTar, and TargetScan. When those miRNAs are screened by two or more tools, they will be considered as potential candidates for upstream regulatory miRNAs of EZH2. Then, a similar method was used to screen upstream lncRNAs. Interaction networks were visualized by Gephi software (https://gephi.org/, accessed on 1 September 2022).

### 2.6. Characterization of Tumor-Infiltrating Immune Cells

Cell type Identification By Estimating Relative Subsets Of RNA Transcripts CIBERSORT) is an analytical tool that relies on RNA-sequencing data or high-throughput arrays to impute the gene expression profiles and estimate the abundances of member cell types in a mixed cell population [20]. We used the latest version of the CIBERSORTx website (https://cibersortx.stanford.edu/, accessed on 10 July 2022) to identify and quantify 22 types of immune cells in tissues based on the leukocyte gene signature matrix file LM22 as the reference [21,22]. These immune cells included 7 distinct types of T cells, naive and memory B cells, plasma cells, natural killer (NK) cells, and myeloid subtypes.

### 2.7. Immune Infiltration Analysis of EZH2

UCSCXenaShiny was used to explore the correlation between the EZH2 and tumor immune cell infiltration. We conducted the analysis based on six distinct quantification methods, including CIBERSORT (abs. mode) [22], “Estimating the Proportion of Immune and Cancer cells” (EPIC) [23], “Microenvironment Cell Populations-counter” (MCP-counter) [24], quanTIseq [25], TIMER [16], and xCell [26]. Immune Cell Abundance Identifier (ImmuCellAI, http://bioinfo.life.hust.edu.cn/ImmuCellAI/ (accessed on 13 July 2022)) is a tool to estimate the abundance of 24 immune cells from gene expression datasets including RNA-Seq and microarray data, which can be applied to estimate the difference of immune cell infiltration in diverse groups [27,28]. We also used ImmuCellAI to compare the difference of tumor-infiltrating immune cells (TICs) in EZH2 high- and low-expression groups.

### 2.8. Correlations between EZH2 Expression Level and Immune-Related Genes, Immune Subtypes, Microsatellite Instability, Tumor Mutation Burden, Tumor Neoantigen Burden, and Mismatch Repair

TISIDB (http://cis.hku.hk/TISIDB/index.php (accessed on 19 July 2022)) is an online platform for evaluating the interaction between tumors and the immune system that integrates data from multiple sources, including results of literature mining from the PubMed database, data from a high-throughput screening for identifying the resistance and sensitivity of tumor cells to T cell-mediated killing, exome and RNA sequencing data for immunotherapy patient cohorts, TCGA, Uniprot, Gene Ontology (GO), and DrugBank [29]. We used TISIDB to conduct the analysis of correlations between EZH2 expression level and immune-related genes and immune subtypes. Microsatellite instability (MSI), tumor mutation burden (TMB), and tumor neoantigen burden (TNB) are all critical tumor markers that could predict the efficacy of immunotherapy [30,31,32]. We used Sangerbox (http://vip.sangerbox.com/home.html, accessed on 19 July 2022), an online bioinformatics analysis tool, to determine the correlations between EZH2 expression level and MSI, TMB, and TNB [33]. TIMER was used to explore the correlations between EZH2 expression and representative mismatch repair (MMR) genes, including MLH1, MLH3, PMS1, PMS2, MSH2, MLH3, MSH6, and EPCAM.

### 2.9. Gene Set Enrichment Analysis and Gene Set Variation Analysis

We first input the EZH2-associated immuno-inhibitor and immuno-stimulator genes obtained in the last section into cBioPortal and selected the TCGA-PRAD dataset to obtain the top 50 co-expressed genes in the total ranking. Then, we used these genes (including EZH2-associated immuno-) as a gene set for enrichment analysis and visualization by using R-package “ggplot2” and “clusterProfiler” as well as online tools STRING (https://string-db.org/, accessed on 2 September 2022) [34], WEB-based GEne SeT AnaLysis Toolkit (WebGestalt, http://www.webgestalt.org/, accessed on 2 September 2022) [35], KEGG Orthology Based Annotation System intelligent version (KOBAS-i, http://kobas.cbi.pku.edu.cn/, accessed on 2 September 2022) [36], and Metascape (http://metascape.org, accessed on 2 September 2022) [37].

Gene Set Variation Analysis (GSVA) was performed by acquiring the gene sets from “H (hallmark gene sets)”, “CP: BIOCARTA (BioCarta gene sets)”, “CP: KEGG (KEGG gene sets)”, “CP: REACTOME (Reactome gene sets)”, and “GO (Gene Ontology gene sets)” in the Molecular Signatures Database (MSigDB, http://www.gsea-msigdb.org/gsea/msigdb/index.jsp (accessed on 5 August 2022)). In addition, we also analyzed two other immune-related gene sets established in previous studies, which were called 7-step Cancer-Immunity Cycle and Immunoscore [38,39].

### 2.10. Immunotherapeutic Response and Drug Sensitivity Prediction

According to RNA-Seq or microarray data, Immunophenoscore (IPS) is an excellent predictor in regard to ICB therapy which can ascertain the immunogenicity determinants and identify the immunological status of the tumor [40]. These determining factors include MHC molecules (MHC), immunomodulators (CP), effector cells (EC), and suppressor cells (SC). Furthermore, the above-mentioned ImmuCellAI can also be employed to predict the response to ICB therapy. IPS and its determinants score can be calculated at https://tcia.at/tools/toolsMain (accessed on 11 August 2022), and ImmuCellAI score was obtained from http://bioinfo.life.hust.edu.cn/ImmuCellAI/ (accessed on 11 August 2022). We used these data to elucidate the association between EZH2 expression and ICB response. Subsequently, the GSE168204 cohort from the Gene Expression Omnibus (GEO) database was used to validate the actual outcome and compare EZH2 expression level as a predictor with the other four ICB response prediction tools by ROC curves.

Computational Analysis of Resistance (CARE) is a computational method that employs compound screen data to determine genome-scale biomarkers of targeted therapeutic response, whose online version is (http://care.dfci.harvard.edu/ (accessed on 15 August 2022)) [41]. The data used for CARE were from three large-scale pharmacogenomic databases, which were Cancer Cell Line Encyclopedia (CCLE), Genomics of Drug Sensitivity in Cancer (GDSC, also named CGP before), and Cancer Therapeutic Response Portal (CTRP) [42,43,44,45]. RNAs associated with Drug (RNAactDrug, http://bio-bigdata.hrbmu.edu.cn/RNAactDrug/ (accessed on 15 August 2022)) is an online database for exploring the relationships of drug sensitivity and RNA molecules, which integrates the data from CellMiner, CCLE, and GDSC [46]. Finally, we analyzed the correlation between EZH2 expression and drug sensitivity or resistance of tumor cells by CARE and RNAactDrug.

### 2.11. Statistical Analysis

The statistical significance of two-group comparisons was determined using unpaired Student’s *t*-test for normally distributed variables, and Mann–Whitney U test for nonnormally distributed variables. To compare more than two experimental groups, the one-way analysis of variance (ANOVA) test was used as a parametric method, and the Kruskal–Wallis test was used as a nonparametric method. The Pearson product–moment correlation coefficient (Pearson’s correlation test) or Spearman’s rank correlation coefficient (Spearman’s correlation test) was used to identify the linear relationship between two groups depending on whether the data were normally distributed. All survival analyses were conducted using K-M product-limit method combined with a log-rank test. The Cox proportional hazard regression model and the nomogram model were established using univariate and multivariate studies. All statistical analyses in this study were performed using R software 3.6.3 (Auckland, New Zealand) and the online tools mentioned above. In all statistical analyses, *p*-values of less than 0.05 were judged to be significant. No significance, *p*-values < 0.05, 0.01, and 0.001 were presented as “ns”, “*”, “**”, and “***”, respectively.

## 3. Results

### 3.1. EZH2 Expression Levels between Tumor and Normal Samples

**Figure 1:**
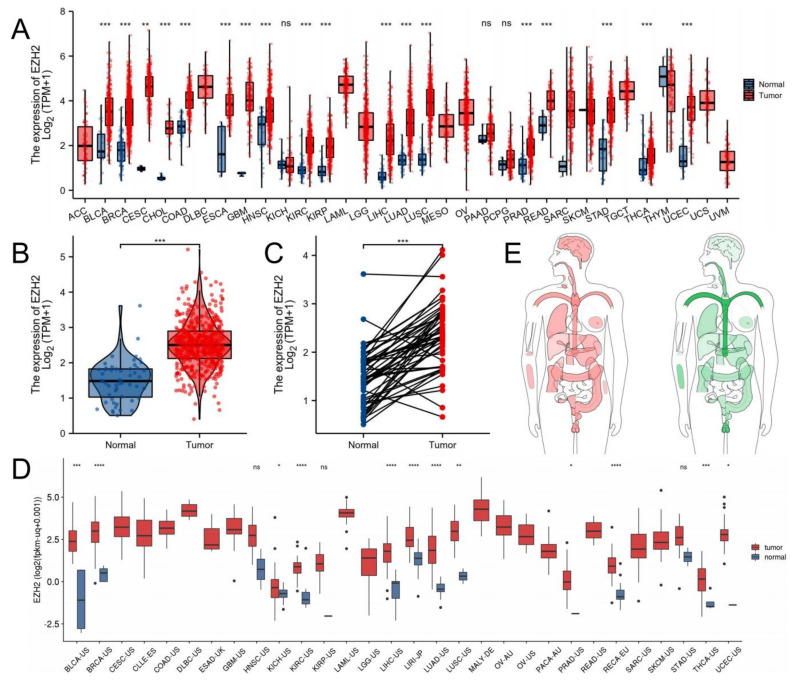
EZH2 mRNA expression levels between tumor and normal samples. EZH2 mRNA expression levels in (**A**) pan-cancer and (**B**) PCa from TCGA database. (**C**) The EZH2 mRNA expression by pairwise boxplot in Pca from TCGA. (**D**) EZH2 mRNA expression levels in pan-cancers from PCAWG. (**E**) Interactive bodymap of EZH2 in humans using GEPIA database. No significance, *p*-values < 0.05, 0.01, 0.001 and 0.0001 were presented as “ns”, “*”, “**”, “***”, and “****”, respectively.

Pan-cancer analysis revealed that EZH2 mRNA expression levels varied across 28 different types of cancer, excluding those for which normal tissue data were unavailable. EZH2 was overexpressed in tumors compared to the normal tissues in most of them, including prostate adenocarcinoma (PRAD) (*p*-value < 0.001, Figure 1A,B). The pairwise boxplot of 52 pairs of PRAD tissues and matched adjacent normal tissues revealed that most of the cancer tissues showed a higher level of EZH2 (*p*-value < 0.001, Figure 1C). We then verified it in PCAWG and obtained the same result (*p*-value < 0.001, Figure 1D). Moreover, we mapped the distribution of EZH2 expression across the human body using the Gene Expression Profiling Interactive Analysis (GEPIA) database (http://gepia.cancer-pku.cn/, accessed on 15 August 2022), which is an interactive web server for analyzing the RNA sequencing expression data of 9736 tumors and 8587 normal samples from the TCGA and the Genotype-Tissue Expression (GTEx) projects [47]. A color scheme where red indicated expression in tumor tissues, green in normal tissues, and the intensity of color indicated the level of expression in each (Figure 1E and Figure S1A).

**Figure 2:**
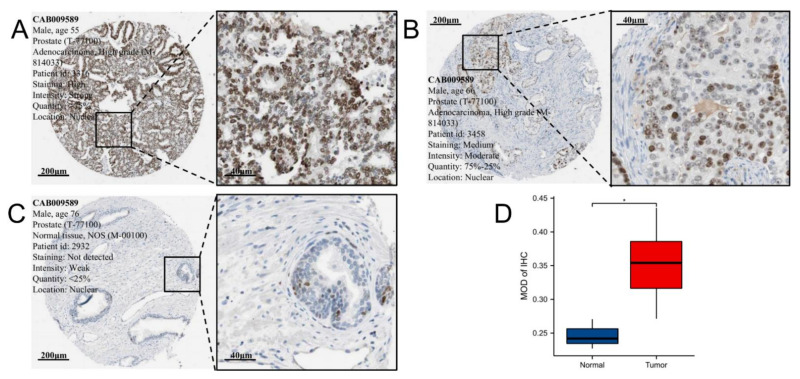
IHC analysis of EZH2. The representative photomicrographs of EZH2 IHC staining with (**A**) strong, (**B**) moderate, and (**C**) weak intensity. (**D**) MOD values of IHC between tumor and normal samples. *p*-values < 0.05 was presented as “*”.

Additionally, we investigated EZH2 protein expression levels in tumor and normal clinical samples by using the HPA database. The representative photomicrographs of EZH2 IHC staining were shown in Figure 2A–C. Quantitative analysis revealed that the MOD values of tumor tissues (n = 12) were significantly higher than those of normal prostate tissues (n = 3) (*p*-value = 0.010, Figure 2D). This suggests that the IHC staining data for EZH2 protein expression are compatible with the transcriptome-level sequencing results.

### 3.2. Associations between EZH2 Expression and Clinicopathologic Parameters and the Prognostic Value of EZH2

**Figure 3:**
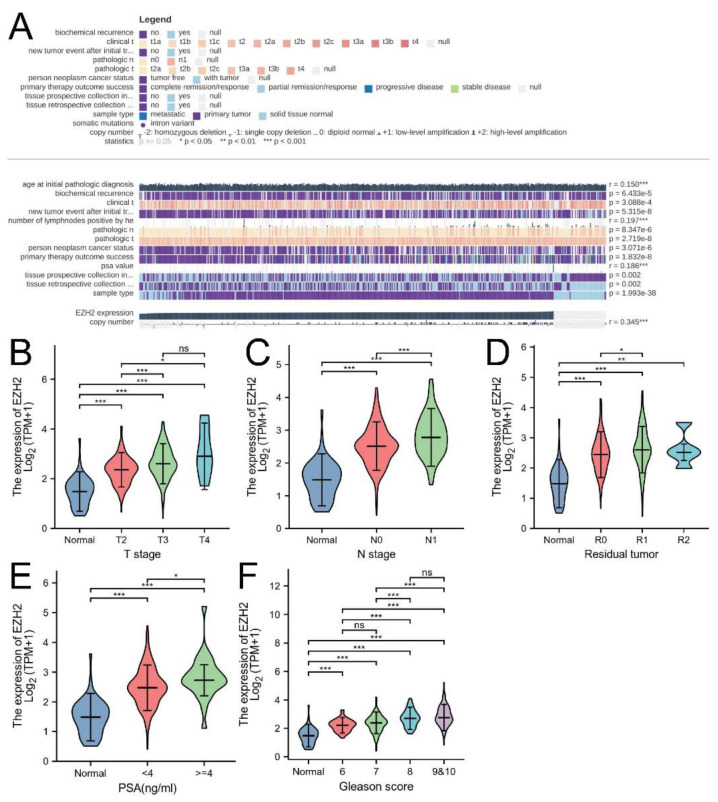
Associations between EZH2 expression and clinicopathologic parameters. (**A**) Associations between EZH2 expression and clinicopathologic parameters using MEXPRESS. Violin plots indicating EZH2 expression in different (**B**) T stage, (**C**) N stage, (**D**) residual tumor, (**E**) PSA, and (**F**) Gleason score from the TCGA database. No significance, *p*-values < 0.05, 0.01, and 0.001 were presented as “ns”, “*”, “**”, and “***”, respectively.

Firstly, we explored the associations between EZH2 expression and clinicopathologic factors by means of MEXPRESS (https://mexpress.be, accessed on 15 August 2022), an online tool that displays the connectivity among TCGA gene expression, DNA methylation, and clinicopathologic data [48,49]. The results showed that EZH2 expression was significantly associated with numerous important clinicopathologic parameters (all *p*-value < 0.01, Figure 3A). T stage, N stage, residual tumor, prostate-specific antigen (PSA) and Gleason score were exhibited individually with violin plots (Figure 3B–F).

**Figure 4:**
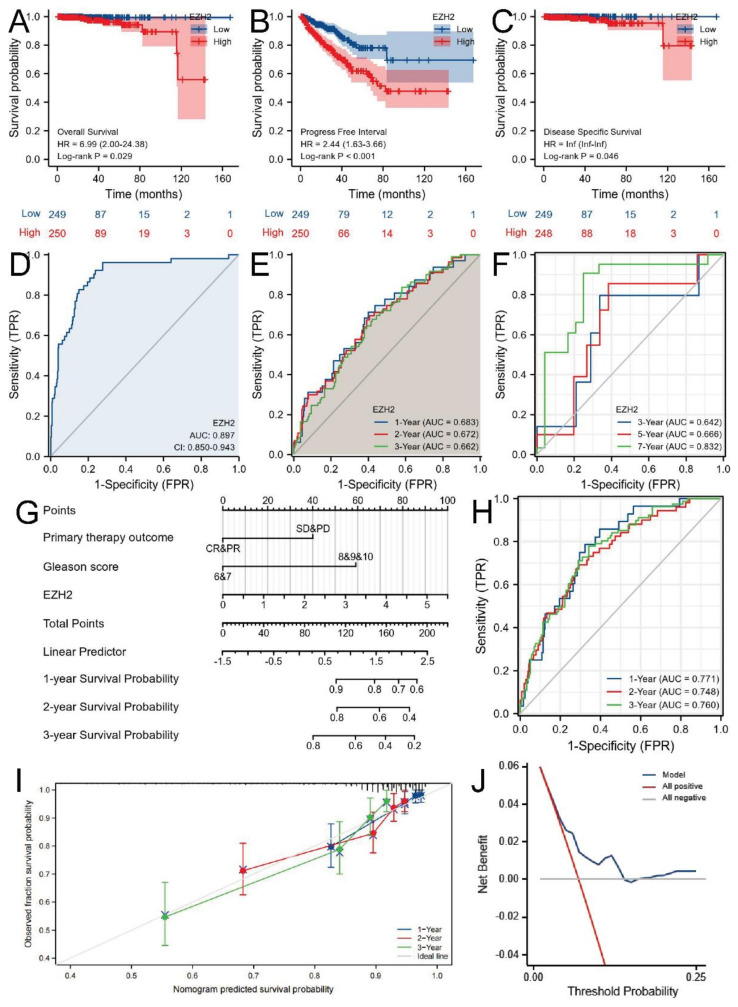
Prognostic value of EZH2 in PCa. The survival curves of EZH2 high- and low-expression groups in (**A**) OS, (**B**) PFS, and (**C**) DSS. (**D**) Diagnostic ROC curve of EZH2. Time-dependent survival ROC curves of (**E**) 1-, 2-, 3-year PFS and (**F**) 3-, 5-, 7-year OS. (**G**) Nomogram and (**H**) ROC curves of 1-, 2-, and 3-year PFS probabilities. (**I**) 1-, 2-, and 3-year PFS calibration curves. (**J**) DCA of prognostic model.

K-M survival analysis showed that the low-expression group has a better prognosis than the high-expression group in OS (log-rank *p*-value = 0.029, Figure 4A), PFS (log-rank *p*-value < 0.001, Figure 4B), and DSS (log-rank *p*-value = 0.046, Figure 4C). The pan-cancer data were also illustrated with forest plots (Supplementary Figure S1B–D). Diagnostic ROC curve analysis demonstrated that EZH2 expression can effectively distinguish tumor and normal tissue (AUC = 0.897, Figure 4D). Time-dependent survival ROC showed that AUC values of 1-, 2-, and 3-year PFS (Figure 4E) and 3-, 5-, and 7-year OS (Figure 4F) were all above 0.64. Therefore, we considered that EZH2 expression has potential as a prognostic factor. Given the clinical significance as well as the long-rank *p*-value, we only developed a prognostic model for PFS in this study. Then, we conducted univariate (Supplementary Figure S1E) and multivariate (Supplementary Figure S1F) Cox regression analyses to determine if EZH2 expression was an independent prognostic factor associated with PFS. We finally concluded that EZH2 expression (HR = 1.513, 95% CI 1.502–2.176, *p*-value = 0.026), primary therapy outcome (HR = 1.813, 95% CI 1.060–3.099, *p*-value = 0.030), and Gleason score (HR = 2.839, 95% CI 1.538–5.240, *p*-value < 0.001) were independent prognostic factors of PFS. We further constructed a nomogram model by integrating these factors to evaluate 1-, 2-, and 3-year PFS probabilities (Figure 4G). The AUC values were 0.771, 0.748, and 0.760, respectively (Figure 4H). Bootstrap validation with 1000 resamples was used to evaluate the model’s accuracy and the possibility of overfitting. The 100-sample bootstrapped calibration curves for the prediction of 1-, 2-, and 3-year PFS were shown in Figure 4I. Finally, DCA was shown in Figure 4J.

### 3.3. Methylation and Genetic Alteration Analysis

**Figure 5:**
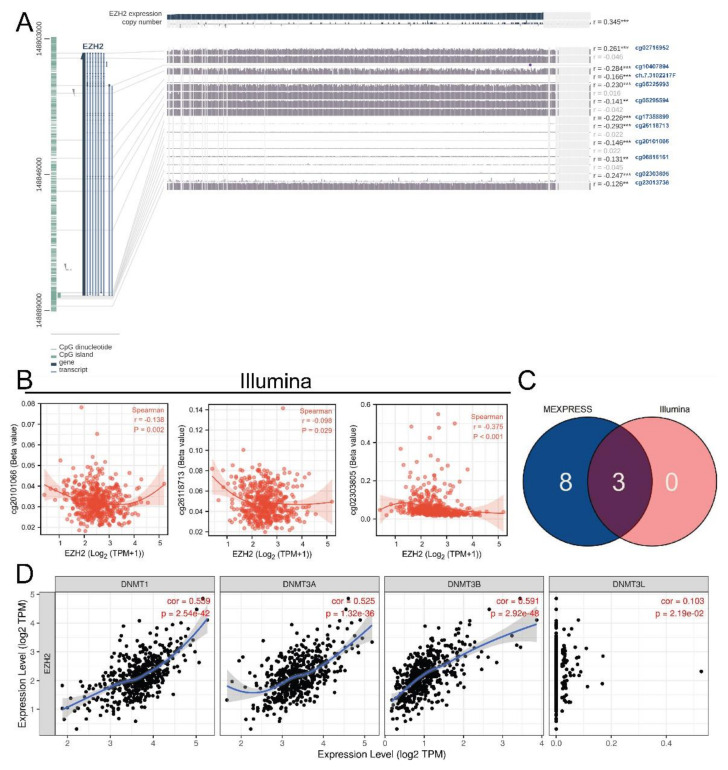
Associations between EZH2 expression and DNA methylation modification. Associations between EZH2 expression and methylation CpG sites in (**A**) MEXPRESS and in (**B**) Illumina. (**C**) Venn diagram indicates the shared methylation CpG sites from MEXPRESS and Illumina. (**D**) Associations between EZH2 expression and DNMTs. *p*-values < 0.01, and 0.001 were presented as “**”, and “***”, respectively.

Eleven methylation CpG sites were associated with EZH2 expression in MEXPRESS (all *p*-value < 0.01, Figure 5A), and 3 in Illumina (all *p*-value < 0.05, Figure 5B). Both sources contained cg20101066 (r = −0.138), cg26118713 (r = −0.098), and cg02303805 (r = −0.375), which were negatively correlated with EZH2 expression, consistent with the probe detection mechanism (Figure 5B,C). In addition, the results showed that there were relatively strong positive correlations between the EZH2 expression and DNMT1, DNMT3A, and DNMT3B (all r > 0.5, *p*-value < 0.001, Figure 5D).

**Figure 6:**
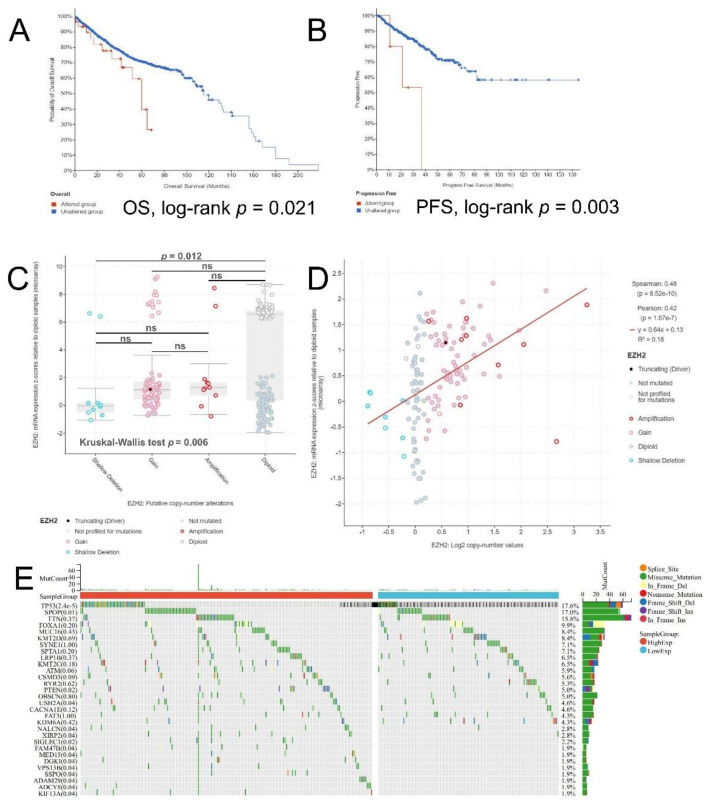
Genetic alteration analysis of EZH2. The survival curves of EZH2 unaltered and altered groups in (**A**) OS and (**B**) PFS. (**C**) Deletion, diploid, copy number gain and amplification are involved in the deregulation of EZH2 expression as analyzed by cBioPortal. (**D**) Association between EZH2 mRNA expression and CNV. (**E**) Waterfall plot shows the mutation landscape by using WGS data from the TCGA-PRAD database.

A total of 10,555 patients/10,962 samples in 25 studies were included in the genetic alteration analysis. EZH2 somatic alteration frequency was 1.8% (altered/profiled), among which, amplification accounted for the majority (Supplementary Figure S2A). The locations of the mutation sites of EZH2 were shown in Supplementary Figure S2B. K-M analysis showed EZH2 unaltered group had a better prognosis than the altered group both in OS (log-rank *p*-value = 0.021, Figure 6A) and PFS (log-rank *p*-value = 0.003, Figure 6B). We also found that diploid, amplification, copy number gain, and shallow deletion all played a role in EZH2 expression deregulation (n = 297). The difference between groups was statistically significant (Kruskal–Wallis test *p*-value = 0.006, Figure 6C). However, after pairwise comparison, only diploid and shallow deletion had a notable difference (Bonferroni–Dunn test *p*-value = 0.012, Figure 6C). In addition, Figure 6D showed that EZH2 copy number was significantly associated with mRNA expression level (n =144; Spearman r = 0.48, *p*-value < 0.001). Finally, we made use of Sangerbox to explore the mutations in driver genes by using whole genome sequencing (WGS) data from the TCGA-PRAD database. We found that TP53 (17.6%), SPOP (17.0%), and TTN (15.8%) were the three genes with the highest mutation rates. Among them, TP53 (*p*-value < 0.001) and SPOP (*p*-value = 0.01) showed significantly high mutation burdens in EZH2 high-expression group (Figure 6E).

### 3.4. Differentially Expressed Genes and Prediction of Upstream miRNAs and lncRNAs of EZH2

**Figure 7:**
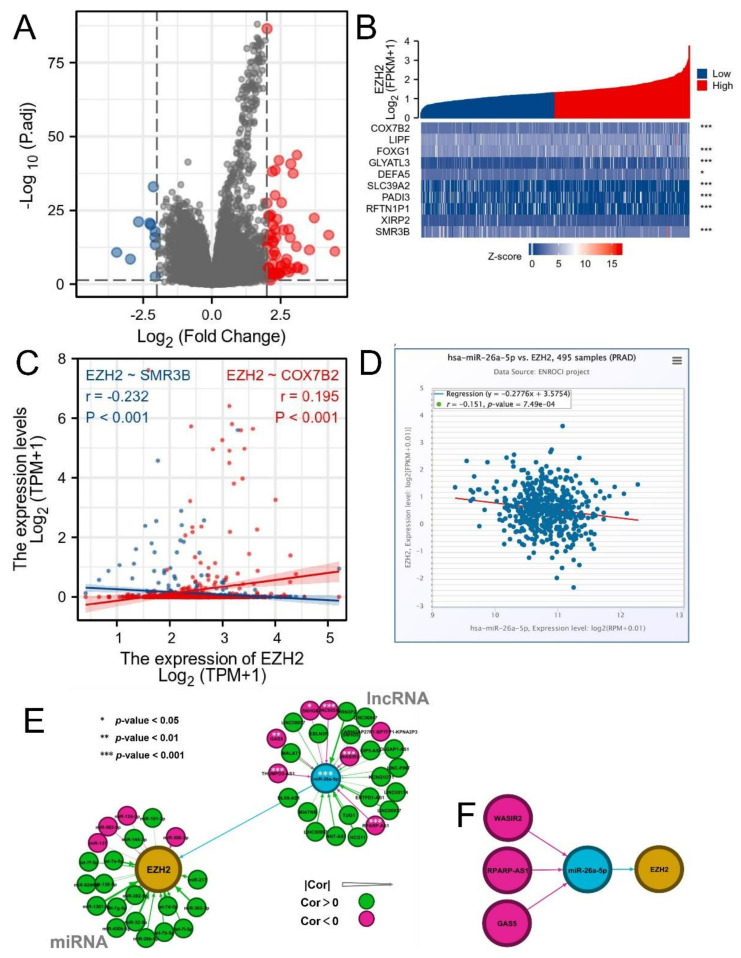
DEGs and prediction of upstream miRNAs and lncRNAs of EZH2: (**A**) Volcano map of DEGs; (**B**) Correlation heat map of the top 5 up- and down-regulated genes with EZH2; (**C**) Correlations of EZH2 with COX7B2 and SMR3B expression in PRAD; (**D**) Correlations of EZH2 with miR-26a-5p in PRAD; (**E**) Network indicates potential upstream miRNAs and lncRNAs that may modulate EZH2; (**F**) The GAS5, THUMPD3-AS1, and WASIR2/miR-26a-5p/EZH2 axis. No significance, *p*-values < 0.05, 0.01, and 0.001 were presented as “ns”, “*”, “**”, and “***”, respectively.

The volcano map of DEGs showed that a total of 61 genes were up-regulated and 10 were down-regulated within the threshold (Figure 7A). Then, we took the top five genes with up- and down-regulation ranked by |log2 FC| value and exhibited them in the correlation heat map (Figure 7B). The correlations of EZH2 with COX7B2 and SMR3B expression in PRAD were further examined (Figure 7C). We found that COX7B2 was positively associated with EZH2 (r = 0.195, *p*-value < 0.001) and SMR3B negatively (r = −0.232, *p*-value < 0.001).

It is generally known that non-coding RNAs (ncRNAs) play an essential role in the regulation of gene expression and the development and progression of cancers. We predicted upstream miRNAs and lncRNAs that may modulate EZH2. 22 miRNAs were initially selected by two or more prediction tools (Supplementary Data S1 Sheet 1). However, in accordance with the mechanism of regulation, miRNA and target gene should be negatively correlated, miR-26a-5p (r = −0.151, *p*-value < 0.001) was the only one left (Figure 7D,E). It was also validated by expression differences and K-M survival analysis. We found that miR-26a-5p was significantly downregulated in PRAD (*p*-value < 0.001, Supplementary Figure S3A) and high-expression of miR-26a-5p was associated with a better PFS (log-rank *p*-value = 0.008, Supplementary Figure S3A). According to these findings, miR-26a-5p was identified as the most potential upstream regulatory miRNA of EZH2. Subsequently, 26 lncRNAs were initially selected in a similar way (Figure 7E). Six of them were negatively correlated with miR-26a-5p and positively correlated with EZH2 (Supplementary Data S1 Sheet 2). We also validated the expression and prognostic differences of these six lncRNAs. Only GAS5, THUMPD3-AS1, and WASIR2 eventually met the qualifications (all *p*-value < 0.001 and log-rank *p*-value < 0.05, Supplementary Figure S3B–D). These results suggested that GAS5, THUMPD3-AS1, and WASIR2 were potential upstream regulatory lncRNAs for the miR-26a-5p/EZH2 axis (Figure 7F).

### 3.5. Proportion and Correlation of Infiltrating Immune Cells in PCa and Normal Tissues

**Figure 8:**
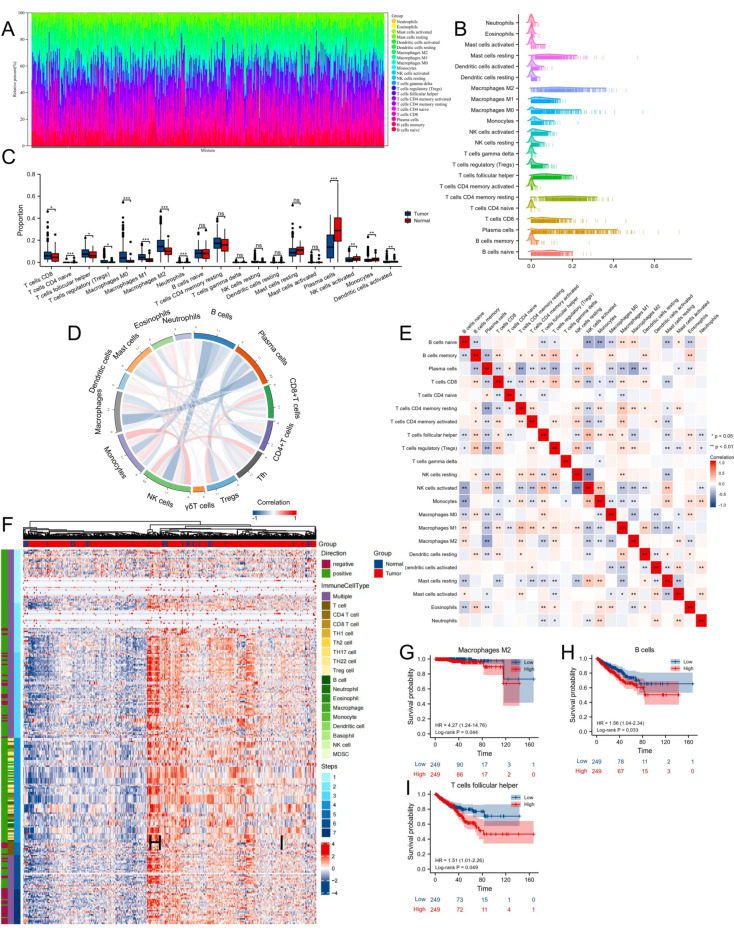
Evaluation of the proportions of immune cell infiltration by CIBERSORTx in TCGA-PRAD cohorts. (**A**) Bar plot and (**B**) ridge plot show the proportion of 22 types of immune cell infiltration. (**C**) Boxplots show the differences in the immune cell distribution between tumor and normal tissues. (**D**) Chord diagram and (**E**) heatmap show the correlation patterns of infiltrating immune cells. (**F**) Heatmap shows the status of anti-cancer immunity across 7-step Cancer-Immunity Cycle. (**G**) The survival curves of macrophages M2 high- and low-infiltration groups in OS. The survival curves of (**H**) B cell and (**I**) Tfh high- and low-infiltration groups in PFS. No significance, *p*-values < 0.05, 0.01, and 0.001 were presented as “ns”, “*”, “**”, and “***”, respectively.

The pattern of the proportion with respect to infiltrating immune cells was shown in Figure 8A,B. After excluding the immune cell types that could not be compared due to their insufficient proportion, we found T cells CD8, T cells CD4 naive, T cells follicular helper (Tfh), T cells regulatory (Tregs), macrophages M0, macrophages M1, macrophages M2, and neutrophils dominated in PCa, while plasma cells, NK cells activated, monocytes, and dendritic cells (DCs) activated dominated in normal tissues (all *p*-value < 0.05, Figure 8C). Correlation patterns of infiltrating immune cells were shown in Figure 8D,E. An anti-cancer immune response can be conceptualized as a series of stepwise events referred to as the cancer-immunity cycle, and we also visualized the status of anti-cancer immunity across a 7-step Cancer-Immunity Cycle based on an established signature set (Figure 8F) [38]. In addition, we classified the patients into two groups according to the median proportion of these TICs. K-M survival analysis indicated that the infiltration level of macrophages M2 was associated with a worse OS (log-rank *p*-value = 0.044, Figure 8G), and the infiltration levels of B cells and Tfh were associated with a worse PFS in PCa (both log-rank *p*-value < 0.05, Figure 8H,I).

### 3.6. Immune Infiltration Analysis of EZH2

The correlations of EZH2 expression and TICs from six methods were shown in Supplementary Figure S4A. A study has systematically evaluated the capabilities and limitations of these computational methods, and we described our results according to the guidelines they gave for method selection (Supplementary Table S1) [50]. We found B cell, T cell CD4+ helper T cell 2 (Th2), T cell CD4+ memory, macrophage, monocyte, and myeloid dendritic cell (mDC) were positively associated with EZH2 mRNA levels (all r > 0, *p*-value < 0.05), while T cell CD4+ helper T cell 1 (Th1), T cell CD8+, NK cell, and endothelial cell were negatively associated with EZH2 mRNA level in PCa (all r < 0, *p*-value < 0.05). Interestingly, the results of cancer-associated fibroblast (CAF) from EPIC and MCP-counter were completely opposite. Likewise, we found B cell, Th1, Th2, macrophage (and macrophage M1), CAF, and mDC were positively associated with EZH2 gene copy number (all r > 0, *p*-value < 0.05), while T cell CD4+, T cell CD4+ non-regulatory, and T cell CD8+ were negatively associated with EZH2 gene copy number in PCa (all r < 0, *p*-value < 0.05) (Supplementary Figure S4B). The results from ImmuCellAI were shown in Supplementary Figure S4C. The correlation between EZH2 expression and biomarkers of immune cells in PCa was shown in Supplementary Table S2.

### 3.7. Correlations between EZH2 Expression Level and Immune-Related Genes, Immune Subtypes, MSI, TMB, TNB, and MMR

**Figure 9:**
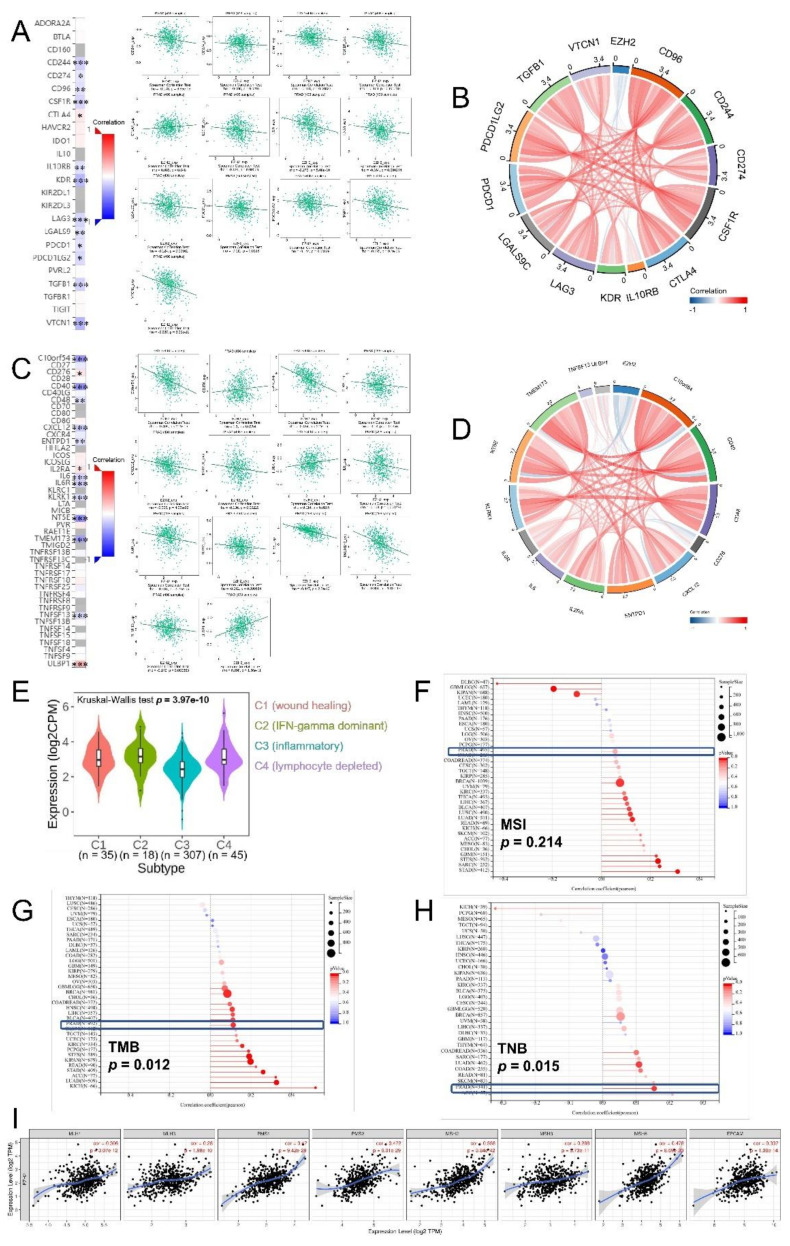
Correlations between EZH2 expression level and (**A**,**B**) immuno-inhibitor genes, (**C**,**D**) immuno-stimulator genes, (**E**) immune subtypes, (**F**) MSI, (**G**) TMB, (**H**) TNB, and (**I**) MMR genes. No significance, *p*-values < 0.05, 0.01, and 0.001 were presented as “ns”, “*”, “**”, and “***”, respectively.

Immune-related genes were categorized into five categories by encoding different functions, including immuno-inhibitor, immuno-stimulator, major histocompatibility complex (MHC), chemokine, and chemokine receptor. We found EZH2 expression level was significantly associated with immuno-inhibitor gene CD96, CD244, CD274, CSF1R, CTLA4, IL10RB, KDR, LAG3, LGALS9, PDCD1, PDCD1LG2, TGFB1, and VTCN1 (all *p*-value < 0.05, Figure 9A,B), and immuno-stimulator gene C10orf54 (VSIR), CD40, CD48, CD276, CXCL12, ENTPD1, IL2RA, IL6, IL6R, KLRK1, NT5E, TMEM173 (STING1), TNFSF13, and ULBP1 (all *p*-value < 0.05, Figure 9C,D). EZH2-associated MHC, chemokine, and chemokine receptor genes were shown in Supplementary Figure S5. In summary, the EZH2 expression level was negatively associated with most of these genes. Subsequently, EZH2 expression differences were observed in four immune subtypes (Figure 9E). TMB and TNB values were positively associated with EZH2 expression, while MSI was not (Figure 9F–H). Figure 9I exhibited that EZH2 expression was strongly positively correlated with the eight MMR genes we selected (r range 0.280–0.558).

### 3.8. EZH2-Associated Enrichment Analysis

**Figure 10:**
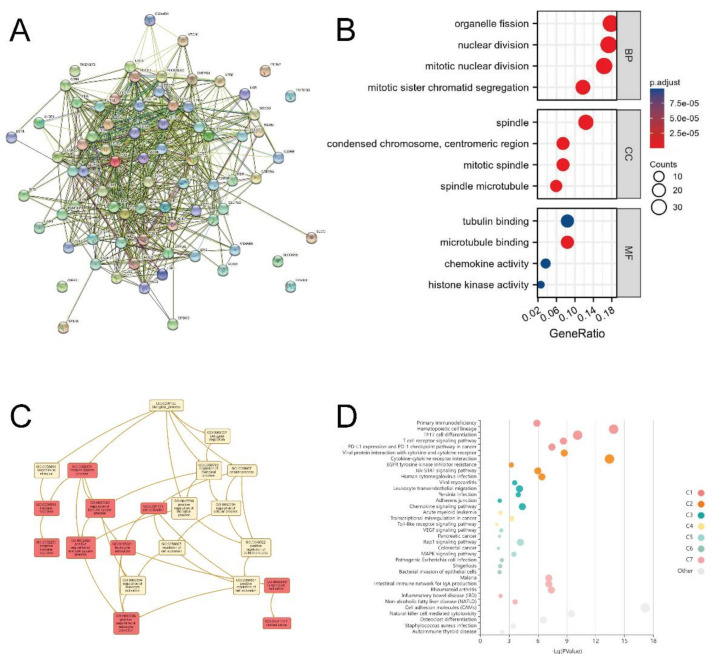
EZH2-associated GSEA: (**A**) PPI network of 13 EZH2-associated immuno-inhibitor genes, 14 EZH2-associated immuno-stimulator genes, and the top 50 co-expressed genes using STRING; (**B**) GO annotation of the abovementioned 77 genes; (**C**) Enriched GO terms network from WebGestalt; (**D**) KEGG and Reactome pathway enrichment analyses of the abovementioned 77 genes using KOBAS-i.

We selected the top 50 co-expressed genes after inputting 13 immuno-inhibitor genes and 14 immuno-stimulator genes into cBioPortal, so that we performed enrichment analysis for a total of 77 genes. The protein–protein interaction (PPI) network of these genes was shown in Figure 10A (Supplementary Data S2 Sheet 1). Figure 10B showed the results of Gene Ontology (GO) enrichment analysis for annotating these genes, and an enriched GO terms network was also plotted (Figure 10C). The results of pathway enrichment analysis in Kyoto Encyclopedia of Genes and Genomes (KEGG), Reactome, PANTHER and WikiPathways cancer were shown in Figure 10D and Figure S6A,B. Based on these findings, we can assume that the T cell receptor (TCR) signaling pathway and JAK-STAT signaling pathway might be related to EZH2-mediated immune events. Furthermore, a comprehensive pathway and process enrichment analysis was also conducted with the following gene ontology sources: KEGG Pathway, GO Biological Processes, Reactome Gene Sets, Canonical Pathways, “the comprehensive resource of mammalian protein complexes” (CORUM), WikiPathways and PANTHER Pathway. Cluster ID was used to color the network of enriched terms, where nodes that share the same cluster ID are typically close together (Supplementary Figure S6C, Supplementary Data S2 Sheet 2).

**Figure 11:**
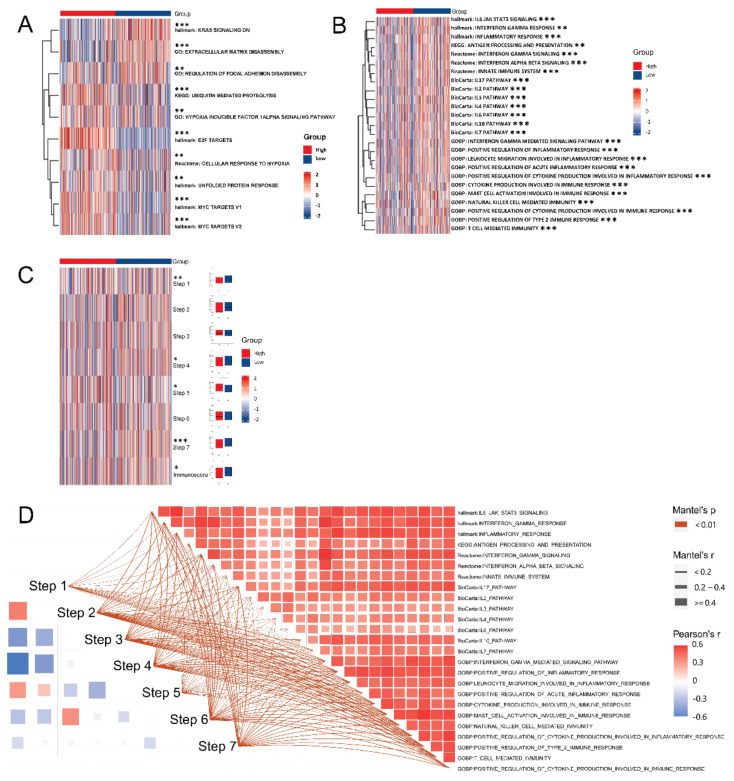
EZH2-associated GSVA: (**A**,**B**) GSVA of hallmark, BioCarta, KEGG, Reactome, and GO gene sets in EZH2 high- and low-expression samples from TCGA-PRAD database; (**C**) GSVA of 7-step Cancer-Immunity Cycle and Immunoscore in EZH2 high- and low-expression samples from TCGA-PRAD database; (**D**) Correlations between and within the MSigDB immune-related biological processes and pathways and 7-step Cancer-Immunity Cycle. *p*-values < 0.05, 0.01, and 0.001 were presented as “*”, “**”, and “***”, respectively.

GSVA results from MSigDB showed significant enrichments in the extracellular matrix, focal adhesion, ubiquitin-mediated proteolysis, hypoxia, targets and pathways in cancer (Figure 11A), as well as several immune-related biological processes and pathways (Figure 11B). Consequently, these findings indicated that EZH2 may play an important role in regulating the occurrence and progression of PCa, as well as triggering immune response. GSVA results from two established gene sets further validated the function of EZH2 in immunity (Figure 11C). Gene lists of two sets were compiled in Supplementary Data S3. Finally, we also explored the connections between and within the MSigDB immune-related biological processes and pathways and the 7-step Cancer-Immunity Cycle by using the Mantel test. The results confirmed that there were more positive connections within the mSigDB immune-related biological processes and pathways than in the 7-step Cancer-Immunity Cycle (Figure 11D).

### 3.9. Immunotherapeutic Response and Drug Sensitivity Prediction

**Figure 12:**
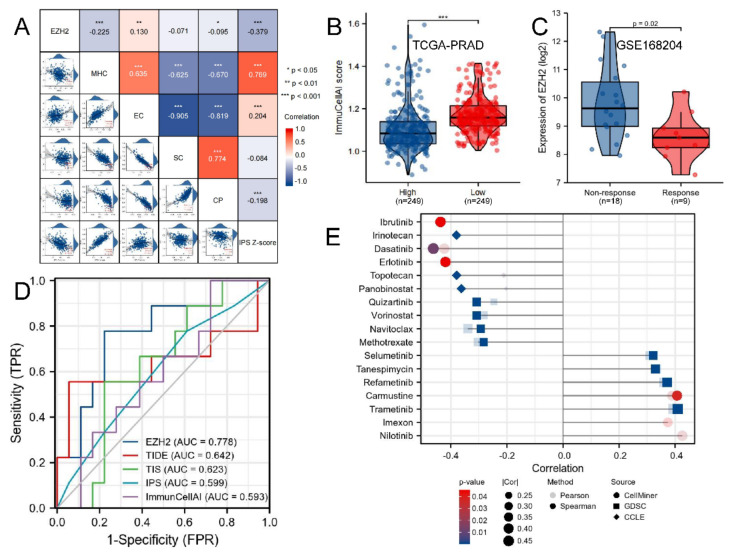
Immunotherapeutic response and drug sensitivity prediction: (**A**) The correlations between EZH2 and MHC, EC, SC, CP, and IPS score in TCGA-PRAD; (**B**) Box-violin plot shows the ImmuCellAI score of EZH2 high- and low-expression groups; (**C**) Box-violin plot shows the distribution of EZH2 for patients with different immunotherapeutic responses in GSE168204 cohort; (**D**) ROC curves of EZH2, TIDE, TIS, IPS, and ImmuCellAI measuring the predictive value about objective response to ICB in GSE168204 cohort; (**E**) Lollipop chart shows the correlation between EZH2 expression and drug sensitivity of tumor cells by RNAactDrug. *p*-values < 0.05, 0.01, and 0.001 were presented as “*”, “**”, and “***”, respectively.

We found EZH2 expression level was negatively associated with the MHC score (r = −0.225, *p*-value < 0.001), CP score (r = −0.095, *p*-value < 0.05), and IPS (r = −0.379, *p*-value < 0.001), while positively associated with EC score (r = 0.130, *p*-value < 0.01) (Figure 12A). The ImmuCellAI scores of the EZH2 high-expression group were significantly lower than those of the low-expression group (*p*-value < 0.001, Figure 12B). In the GEO dataset, EZH2 expression levels in non-response to the ICB group (n = 18) were significantly higher than the response group (n = 9) (*p*-value = 0.020, Figure 12C). In addition to IPS and ImmuCellAI, we also plotted the ROC curves of tumor immune dysfunction and exclusion (TIDE, http://tide.dfci.harvard.edu/, accessed on 15 August 2022) and T-cell inflammatory signature (TIS, gene list was in Supplementary Data S4), which were also outstanding ICB therapy prediction algorithms [51,52]. Figure 12D exhibited that the AUC values of EZH2, TIDE, TIS, IPS, and ImmuCellAI were 0.778, 0.642, 0.623, 0.599, and 0.593, respectively. In summary, we concluded that EZH2 expression level reflects the immune status of patients well and predicts the response to ICB therapy.

By analyzing using RNAactDrug, we found that the sensitivities of Nilotinib, Imexon, Trametinib, Carmustine, Refametinib, Tanespimycin, and Selumetinib (Supplementary Figure S7A) were positively associated with EZH2 expression level, while the sensitivities of Ibrutinib, Irinotecan, Dasatinib, Erlotinib, Topotecan, Panobinostat, Quizartinib, Vorinostat, Navitoclax, and Methotrexate (Supplementary Figure S7B) negatively (Figure 12E, complete compound list was in Supplementary Data S5 Sheet 1). The selected compounds from CARE were listed in Supplementary Data S5 Sheets 2–4, among which, Nilotinib was validated as one of the most promising candidates (Supplementary Data S5 Sheet 5, Supplementary Figure S7C).

## 4. Discussion

Under the circumstance of gene transcription level being mediated by chromatin, the process is defined as epigenetics rather than genetics since no alterations are made to the DNA sequence. It is widely accepted that EZH2-mediated histone methylation is a crucial epigenetic regulatory mechanism. Based on the location and condition of methylation, histone lysine residue methylation can either stimulate or inhibit gene transcription [53]. H3K27me3 is associated with transcriptional inhibition, while EZH2 catalyzes H3K27me3, hence suppressing target gene expression. Many of these genes can suppress tumorigenesis. In the present study, we found that EZH2 was highly expressed in a range of malignancies and that the expression of EZH2 in PCa tissues was higher than in normal prostate tissues. To further investigate the relationship between EZH2 and PCa, we classified the prostate cancer patients into high-expression and low-expression groups based on the median EZH2 expression levels of all patients. We found that patients with high EZH2 expression had worse OS, PFS, and DSS than those with low EZH2 expression. In addition, high EZH2 expression was correlated with age, T-stage, N-stage, initial treatment outcome, high grade, PCa type, PSA value, and Gleason score. Using univariate and multivariate Cox regression analysis, we determined that EZH2 can be an independent prognostic factor for prostate cancer. Additionally, in order to facilitate physicians in predicting the prognosis of EZH2 patients, we created a prediction nomogram based on EZH2 expression levels and associated clinicopathological factors, and with a relatively satisfactory performance. Consequently, it is reasonable to assume that EZH2 may serve as a predictor of clinical outcome in patients with PCa.

To have a deeper understanding regarding the function of EZH2 in PCa, we categorized patients based on their EZH2 expression level and identified DEGs between the high and low EZH2 expression groups. These DEGs were shown to be crucial in regulating the development and progression of several tumors. FOXG1 mediates cancer cell metastasis via the Wnt/β-catenin pathway in hepatocellular carcinoma (HCC) cells and predicts the outcome of HCC following surgery [54]. PADI3 promotes cell cycle arrest via Sirt2-AKT-p21 and functions as a tumor suppressor gene in colon cancer [55]. It is well known that miRNAs can exert regulatory effects on target mRNAs by destabilizing them and inhibiting their translation. Through online prediction as well as correlation, expression, and survival analysis screening, we finally identified miR-26a-5p as the most potential upstream miRNA of EZH2, which is also consistent with a previous study [56]. Similarly, it has been shown that WASIR2 knockdown suppressed the malignant activity of osteosarcoma cells; RPARP-AS1 and GAS5 can be used as prognostic biomarkers for lung adenocarcinoma (LUAD) and PCa, respectively [57,58,59].

Tumorigenesis is not only determined by gene alterations or epigenetic changes in cancer cells but also by tumor microenvironment (TME) [60]. The TME is typically comprised of fibroblasts, pericytes, immune cells, and endothelial cells. During tumor progression, each cell type interacts dynamically with cancer cells to alter the surrounding environment into one that promotes tumor development. These alterations consist of the recruitment of fibroblasts, migration of immune cells, stroma remodeling, and progression of tumor-specific vasculature [61]. The prognosis of cancer is correlated with immune cell infiltration and activation, thus the TME, which is composed of diverse infiltrating immune and stromal cells, may greatly impact therapy response and clinical outcome, in addition to playing a crucial role in cancer etiology and progression [62,63]. This study integrated multiple prediction methods to assess the association between EZH2 expression and the abundance of infiltrating immune cells in PCa in order to better determine the impact of the tumor immune microenvironment. We observed a positive association between EZH2 expression and the abundance of B cell, Th2, T cell CD4+ memory, macrophage, monocyte, and mDC, but a negative correlation with the infiltration degree of Th1, T cell CD8+, NK cell, and endothelial cell. Consequently, EZH2 may indirectly play a crucial role in the remodeling of the immune microenvironment.

In addition to immune cells, other components of the TME play an essential role. CAFs are one of the most prevalent cells in the TME, and their composition of distinct clusters confers functional variability [64]. These CAF clusters release an assortment of cytokines with various immunomodulatory characteristics. Some have tumor-suppressing properties, whereas others have tumor-promoting ones [65,66]. In our study, the two recommended methods revealed contradictory results regarding the association between EZH2 expression and CAF abundance. We speculated that this situation might be explained by the heterogeneity of CAF.

We analyzed the co-expression correlation between EZH2 and the genes encoding immuno-inhibitor, immuno-stimulator, MHC, chemokine, and chemokine receptor. The results revealed that EZH2 expression is associated with a variety of genes involved in the immune system. This suggested that EZH2 may have a close relationship with the regulation of antigen recognition, immune cell recruitment, and transport capacity [67]. In addition, EZH2 was associated with the expression of well-known targets such as CD274 (PD-L1), CTLA4, LAG3, PDCD1 (PD-1), and PDCD1LG2 (PD-L2). Previous studies have demonstrated that TMB, TNB, and MMR are all significantly associated with sensitivity to immunotherapy [31,32,68]. In the present study, EZH2 expression was also significantly correlated with TMB and TNB values as well as the expression of various MMR-related genes in PCa. In summary, all these results indicated that the currently approved immunotherapy in the treatment of PCa might be enhanced by inhibiting EZH2.

In terms of potential regulatory mechanisms, enrichment analyses such as GO and KEGG suggested that EZH2 was strongly associated with cell division and immune response regulation, and the immune response mediated by EZH2 may entail the TCR signaling pathway and JAK-STAT signaling pathway. It has been reported that, when EZH2 was knocked down in PCa, IFNGR1 expression was restored, and when IFN therapy was applied, significant activation of IFN-JAK-STAT1 tumor suppressor signaling occurred [69]. Another study showed that by demethylating H3K27me3 marks in CAFs, pro-inflammatory cytokine-driven EZH2 downregulation maintains the senescence-associated secretory phenotype and promotes peritoneal tumor progression of gastric cancer (GC) via JAK-STAT signaling [70]. Similarly, based on the results of the GSVA, we found that multiple pathways related to tumorigenesis, progression, and immune activation were enriched, including the IL-6-JAK-STAT3 pathway. The 7-step Cancer-Immunity Cycle reflects the anticancer immune response. They include Step 1: release of cancer cell antigens; Step 2: cancer antigen presentation; Step 3: priming and activation; Step 4: trafficking of immune cells to tumors; Step 5: infiltration of immune cells into tumors; Step 6: recognition of cancer cells by T cells; and Step 7: the killing of cancer cells [38]. We found Steps 1, 4, and 7 were enriched in the EZH2-low group, suggesting that the EZH2-high group is more prone to immunosuppression and affects the function of immune cells in PCa.

Although an integrated analysis of multi-omics data has provided some encouraging clinical translation findings in this study, these findings should be interpreted within the context of the limitations. Foremost, this study may be subject to systematic bias because multiple information sources were obtained and analyzed from different databases. Then, the TCGA database comprises only the PRAD cohort, which, despite its predominance in the pathological types of PCa, is still not completely representative of PCa. Thirdly, we lack prospective clinical cohort studies validating the prognostic value of EZH2 for response to immunotherapy for PCa. Lastly, due to the prevalence of COVID-19 and the territorial policy for the prevention of epidemics, access to the laboratory was extremely difficult, thus only bioinformatics analysis was performed in this study. We will explore the detailed mechanisms of EZH2 regulation regarding the tumor immune microenvironment in vitro and in vivo systematically in the near future.

## 5. Conclusions

In conclusion, our study demonstrates that EZH2 could be a potentially efficient predictor of prognosis and immune response in PCa patients. There is also evidence suggesting that EZH2 expression in PCa has been associated with DNA methylation modifications, TME, immune-related genes, TMB, TNB, and MMR. These findings expand the understanding of EZH2 in PCa therapy and provide new insights into its immunological applications. It is reasonable to assume that EZH2 could be a practical target for immunotherapy in PCa patients. These findings will benefit the precision treatment of patients with PCa and have significant clinical consequences.

## Data Availability

Research data are stored in an institutional repository and will be shared upon reasonable request to the first author Tian-Qi Du (dutq21@lzu.edu.cn).

## Supplementary Materials

The following supporting information can be downloaded at: https://www.mdpi.com/article/10.3390/biom12111617/s1, Figure S1. (A) Boxplots show EZH2 mRNA expression levels in pan-cancer from TCGA and GTEx databases. Forest plots show the prognostic value of EZH2 expression in pan-cancer, employing (B) OS, (C) PFS, and (D) DSS as outcome endpoints. (E) Univariate and (F) multivariate cox hazard regression analyses in PCa from TCGA database. Figure S2. (A) Summary of alteration on a query of EZH2 from cBioPortal. (B) The locations of the mutation sites of EZH2. Figure S3. Expression analysis and PFS analysis of (A) miR-26a-5p, (B) GAS5, (C) THUMPD3-AS1, and (D) WASIR2 in PRAD patients. Figure S4. (A) Correlations between EZH2 expression and TICs using CIBERSORT (abs. mode), EPIC, MCP-counter, quanTIseq, TIMER, and xCell. (B) Correlations between EZH2 copy number and TICs using CIBERSORT (abs. mode), EPIC, MCP-counter, quanTIseq, TIMER, and xCell. (C) Boxplots show TICs in EZH2 high- and low-expression groups from ImmuCellAI. Figure S5. Correlations between EZH2 expression level and (A,B) MHC genes, (C,D) chemokine genes, and (E,F) chemokine receptor genes. Figure S6. (A) PANTHER and (B) WikiPathways cancer pathway enrichment analyses of 13 EZH2-associated immuno-inhibitor genes, 14 EZH2-associated immuno-stimulator genes, and the top 50 co-expressed genes using. WebGestalt. (C) Network shows a comprehensive pathway and process enrichment analysis of the abovementioned 77 genes using Metascape. Figure S7. Correlations between EZH2 expression and drug (A) sensitivity and (B) resistance of tumor cells by RNAactDrug. (C) Venn diagram indicates the shared drug of three source using CARE.
