# Supplementary material for: EZH2 as a Prognostic Factor and Its Immune Implication with Molecular Characterization in Prostate Cancer: An Integrated Multi-Omics in Silico Analysis
Source: Biomolecules. 2022 Nov 2;12(11):1617. doi: 10.3390/biom12111617 (PMC9687944; doi:10.3390/biom12111617)
Supplement: Supplementary file 1 [file biomolecules-12-01617-s001.zip › Supplementary Fig. S1 R2.pdf]

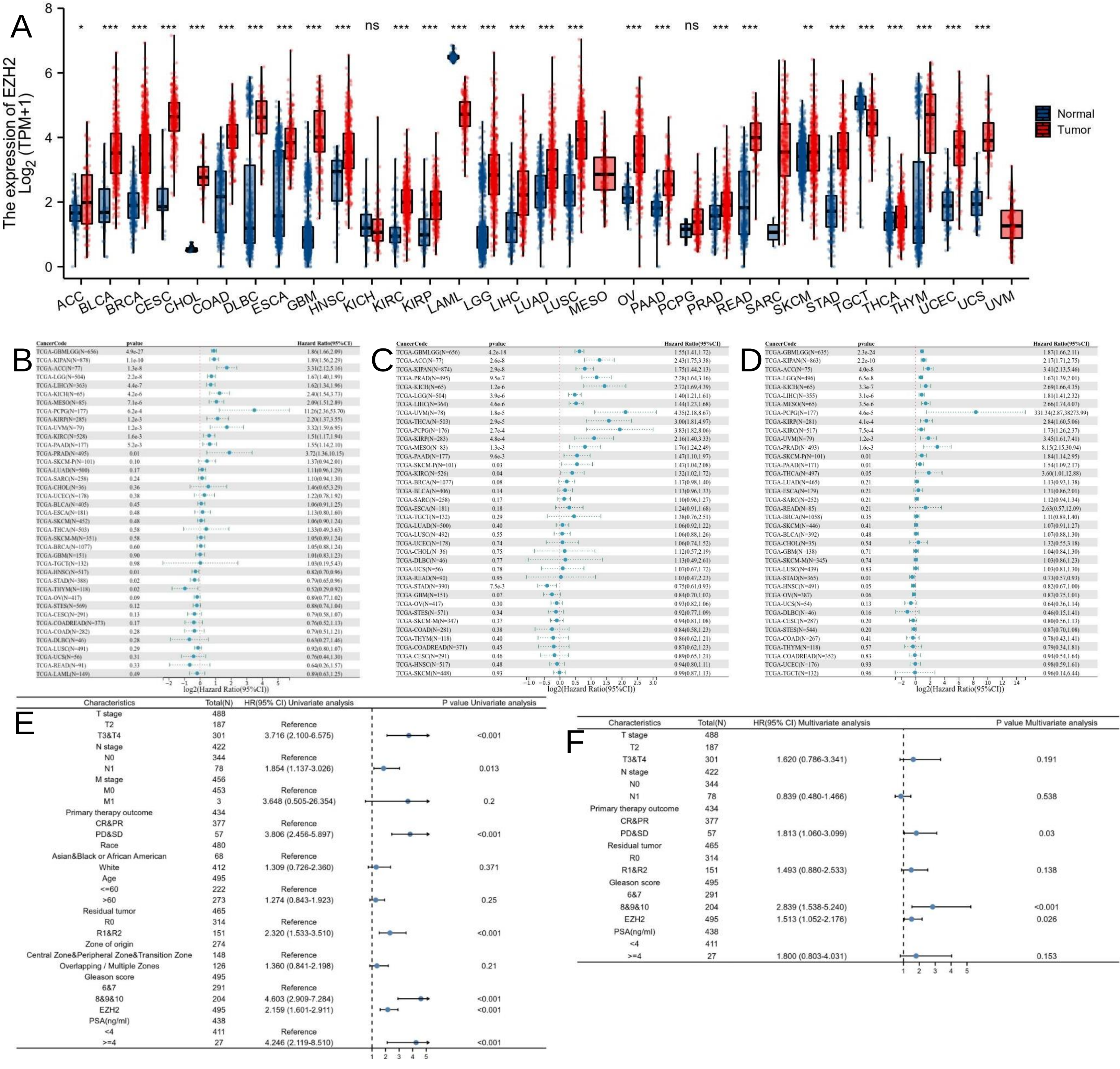

Figure S1. (A) Boxplots show EZH2 mRNA expression levels in pan-cancer from TCGA and GTEx databases. Forest plots show the prognostic value of EZH2 expression in pan-cancer, employing (B) OS, (C) PFS, and (D) DSS as outcome endpoints. (E) Univariate and (F) multivariate cox hazard regression analyses in PCa from TCGA database.
