# Supplementary material for: EZH2 as a Prognostic Factor and Its Immune Implication with Molecular Characterization in Prostate Cancer: An Integrated Multi-Omics in Silico Analysis
Source: Biomolecules. 2022 Nov 2;12(11):1617. doi: 10.3390/biom12111617 (PMC9687944; doi:10.3390/biom12111617)
Supplement: Supplementary file 1 [file biomolecules-12-01617-s001.zip › Supplementary Fig. S2 R2.pdf]

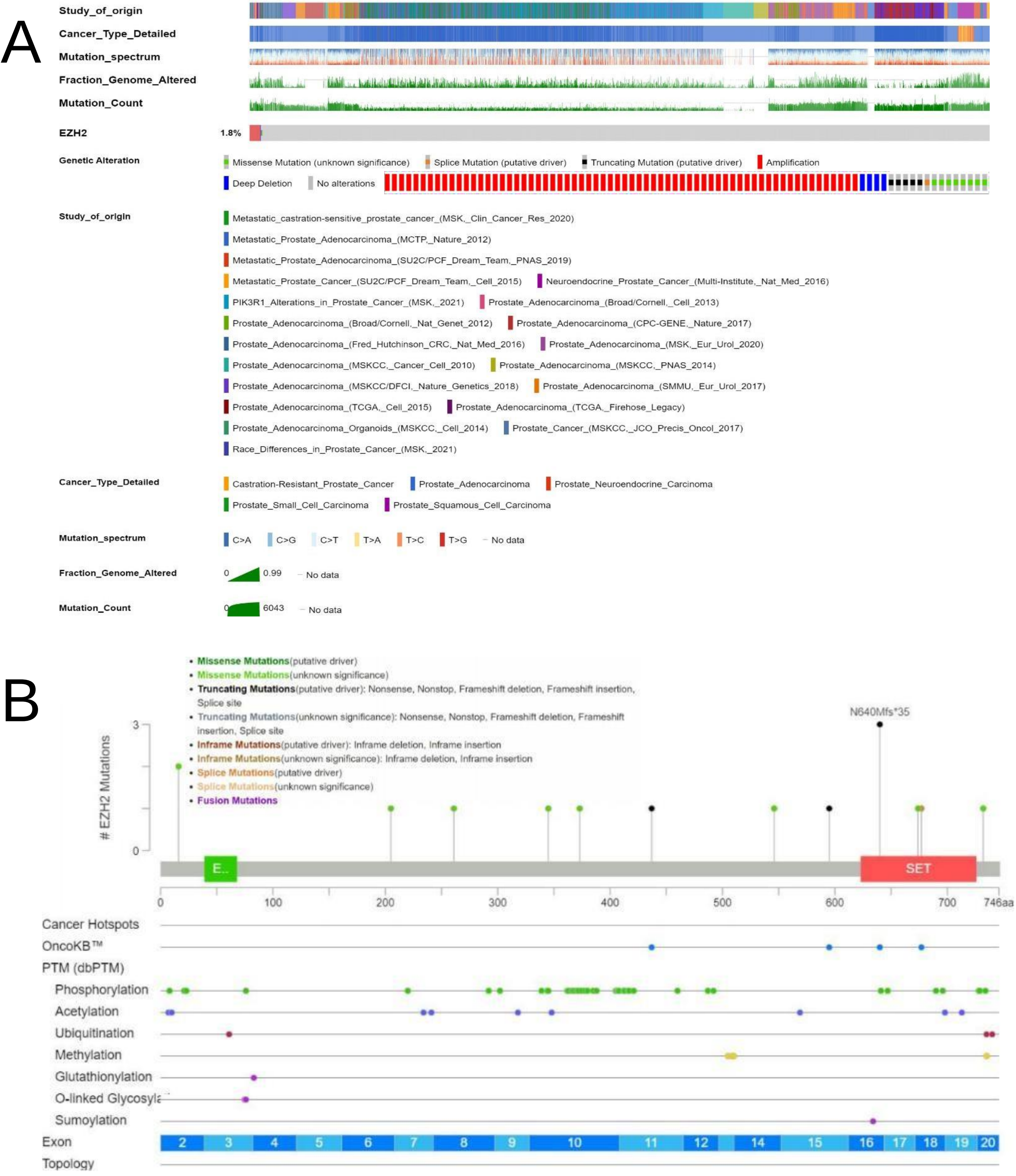

Figure S2. (A) Summary of alteration on a query of EZH2 from cBioPortal. (B) The locations of the mutation sites of EZH2.
