# Supplementary material for: EZH2 as a Prognostic Factor and Its Immune Implication with Molecular Characterization in Prostate Cancer: An Integrated Multi-Omics in Silico Analysis
Source: Biomolecules. 2022 Nov 2;12(11):1617. doi: 10.3390/biom12111617 (PMC9687944; doi:10.3390/biom12111617)
Supplement: Supplementary file 1 [file biomolecules-12-01617-s001.zip › Supplementary Fig. S6 R2.pdf]

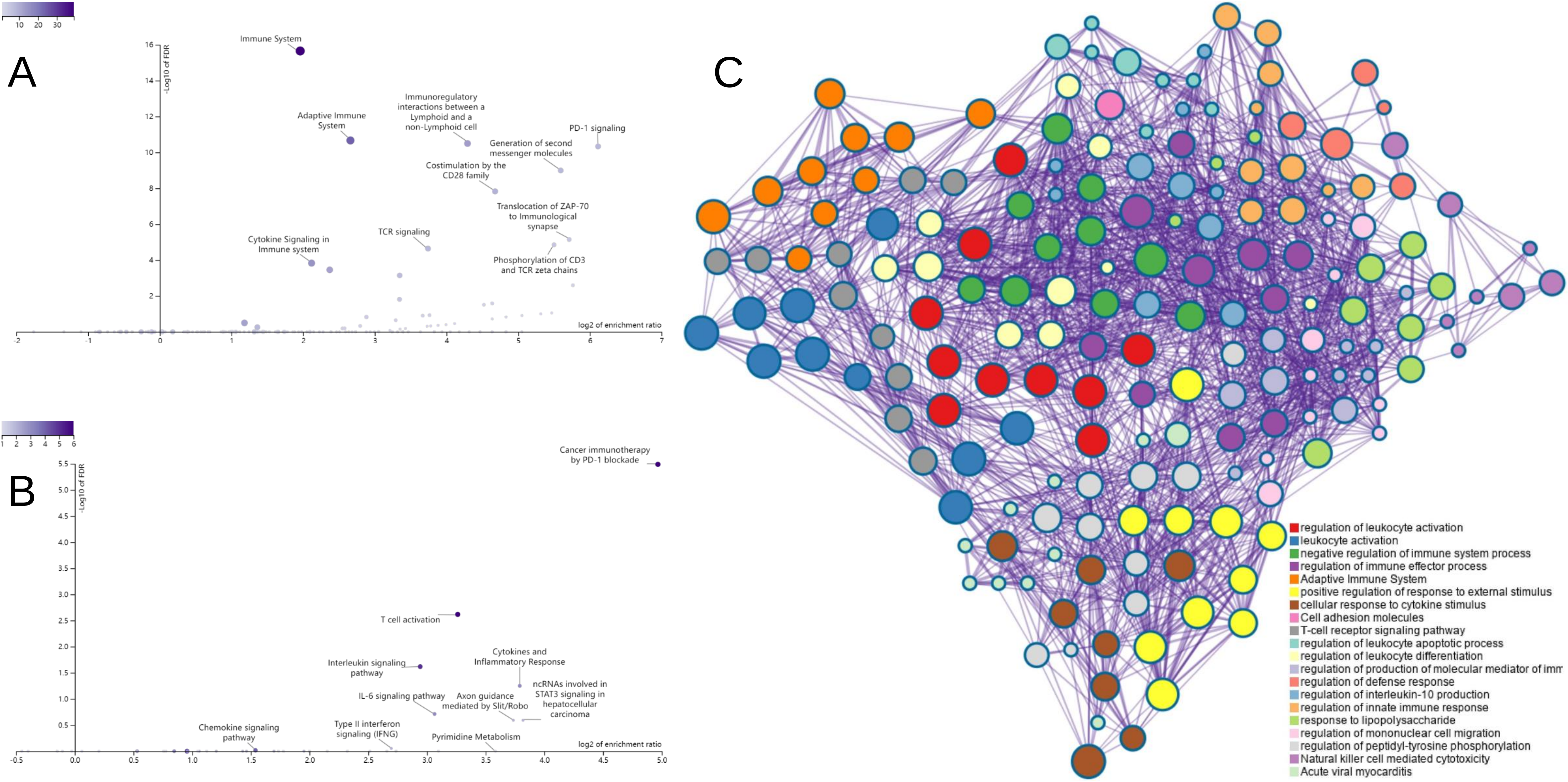


Figure S6. (A) PANTHER and (B) WikiPathways cancer pathway enrichment analyses of 13 EZH2-associated immuno-inhibitor genes, 14 EZH2-associated immuno-stimulator genes, and the top 50 co-expressed genes using. WebGestalt. (C) Network shows a comprehensive pathway and process enrichment analysis of the abovementioned 77 genes using Metascape.
