# Supplementary material for: EZH2 as a Prognostic Factor and Its Immune Implication with Molecular Characterization in Prostate Cancer: An Integrated Multi-Omics in Silico Analysis
Source: Biomolecules. 2022 Nov 2;12(11):1617. doi: 10.3390/biom12111617 (PMC9687944; doi:10.3390/biom12111617)
Supplement: Supplementary file 1 [file biomolecules-12-01617-s001.zip › Supplementary Fig. S7 R2.pdf]

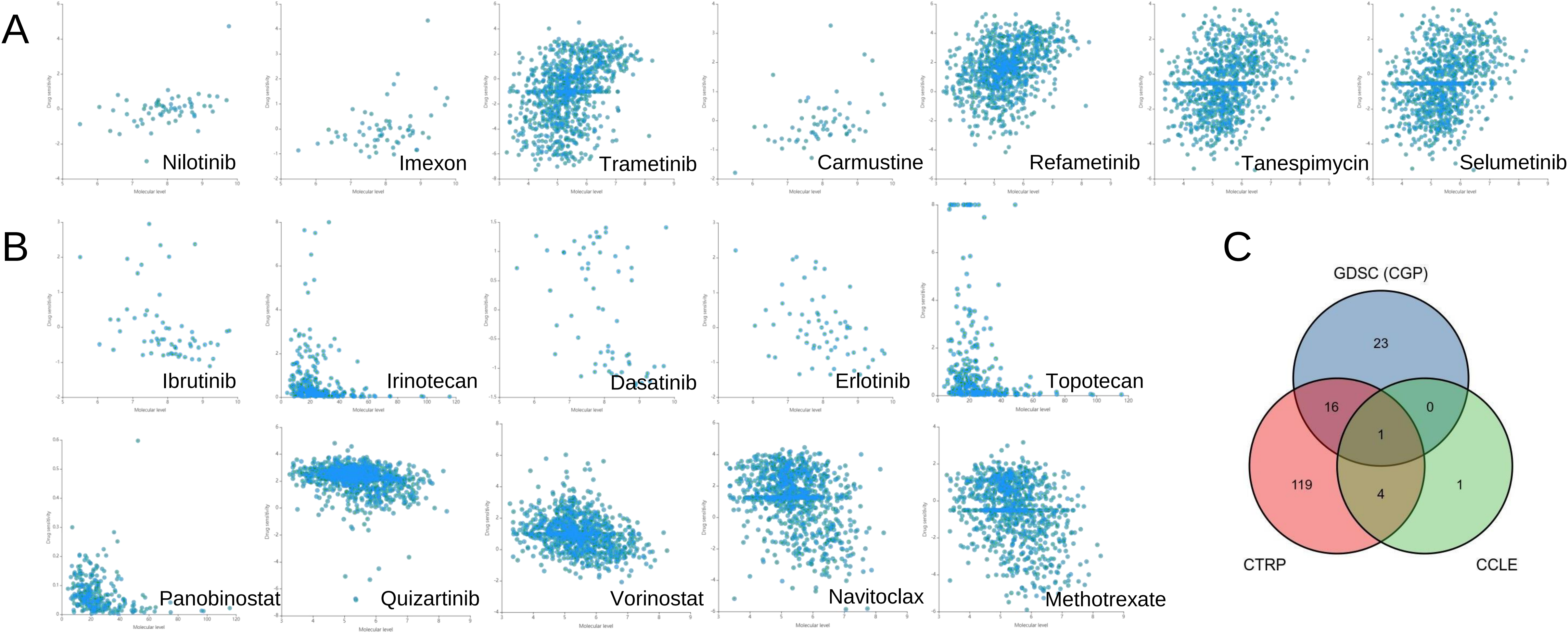

Figure S7. Correlations between EZH2 expression and drug (A) sensitivity and (B) resistance of tumor cells by RNAactDrug. (C) Venn diagram indicates the shared drug of three source using CARE.
